# ORGANIZATIONAL CONTEXT'S EFFECT ON CARE AIDES' PSYCHOLOGICAL EMPOWERMENT IN WESTERN CANADA

**DOI:** 10.1093/geroni/igac059.1241

**Published:** 2022-12-20

**Authors:** Alba Iaconi, Yinfei Duan, Yuting Song, Matthias Hoben, Leslie Hayduk, Peter Norton, Carole Estabrooks

**Affiliations:** University of Alberta, Edmonton, Alberta, Canada; University of Alberta, Edmonton, Alberta, Canada; Qingdao University, Qingdao, Shandong, China (People's Republic); University of Alberta, Edmonton, Alberta, Canada; University of Alberta, Edmonton, Alberta, Canada; University of Calgary, Calgary, Alberta, Canada; University of Alberta, Edmonton, Alberta, Canada

## Abstract

This quantitative cross-sectional sub project investigated the effects of organizational context and individual characteristics on psychological empowerment of care aides working in nursing homes. We analyzed data collected from 3765 care aides from 91 nursing homes across Western Canada between 09/2019 and 03/2020. From the random-intercept mixed effects regression models we identified significant predictors at different levels for each component of psychological empowerment. At the organizational outer context level: region and home ownership model. At the inner context (care unit) level: formal interactions (β=-0.07, p=0.03; competence), evaluation (β=0.20, p<0.02; self-determination), culture (β=0.20, p<0.02; self-determination), communication (β=0.16, p<0.001; self-determination), and social capital (β=0.22, p=0.01; impact). At the individual level: care aides’ sex, language and job satisfaction.These findings suggest important ways in which contextual elements may influence staff quality of work life characteristics and underscore the need to consider context operating at different levels, as well as consider individual and contextual interaction.

